# The gut microbiota, a key to understanding the health implications of micro(nano)plastics and their biodegradation

**DOI:** 10.1111/1751-7915.14182

**Published:** 2022-11-22

**Authors:** Cristina Jiménez‐Arroyo, Alba Tamargo, Natalia Molinero, M. Victoria Moreno‐Arribas

**Affiliations:** ^1^ Instituto de Investigación en Ciencias de la Alimentación (CIAL) CSIC‐UAM Madrid Spain

## Abstract

The effects of plastic debris on the environment and plant, animal, and human health are a global challenge, with micro(nano)plastics (MNPs) being the main focus. MNPs are found so often in the food chain that they are provoking an increase in human intake. They have been detected in most categories of consumed foods, drinking water, and even human feces. Therefore, oral ingestion becomes the main source of exposure to MNPs, and the gastrointestinal tract, primarily the gut, constantly interacts with these small particles. The consequences of human exposure to MNPs remain unclear. However, current in vivo studies and in vitro gastrointestinal tract models have shown that MNPs of several types and sizes impact gut intestinal bacteria, affecting gut homeostasis. The typical microbiome signature of MNP ingestion is often associated with dysbiosis and loss of resilience, leads to frequent pathogen outbreaks, and local and systemic metabolic disorders. Moreover, the small micro‐ and nano‐plastic particles found in animal tissues with accumulated evidence of microbial degradation of plastics/MNPs by bacteria and insect gut microbiota raise the issue of whether human gut bacteria make key contributions to the bio‐transformation of ingested MNPs. Here, we discuss these issues and unveil the complex interplay between MNPs and the human gut microbiome. Therefore, the elucidation of the biological consequences of this interaction on both host and microbiota is undoubtedly challenging. It is expected that microbial biotechnology and microbiome research could help decipher the extent to which gut microorganisms diversify and MNP‐determinant species, mechanisms, and enzymatic systems, as well as become important to understand our response to MNP exposure and provide background information to inspire future holistic studies.

## Graphical abstract

Growing evidence implicates the gut microbiome as a key challenge for the connections between ingested MNPs and human health. MPs may suffer fragmentation and biotransformation during the digestive transit. The gut microbiome affects the host and could also affect the bioaccumulation of MNPs in the human body; therefore, the extent of MNPs gut/health effects and their microbial metabolic fate is reviewed and discussed.

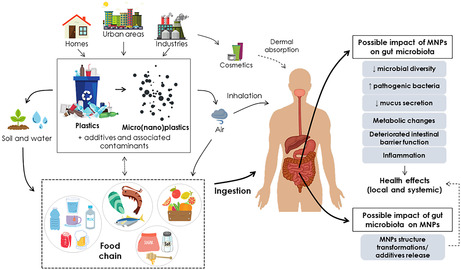

## GENERAL FEATURES

For decades, plastic use has been popularized in industries and domestically and has become indispensable in all aspects of human endeavors. Therefore, plastic production has tripled in the last 25 years, with a global production that reached 367 million tons in 2020, with 55 million tons produced in Europe, and approximately a third of the total produced in China (Plastics Europe, 2021).

Plastic residues comprise numerous types of polymers with different degrees of wear, shape, and size (SAPEA, 2019), which can also break into smaller particles through physical, chemical, biological mechanisms, and/or their combination (Zettler et al., 2013). Size is one of the most used criteria to classify plastic waste due to its ecological relevance, usually classified into three main groups: macro‐ (>25 mm), meso‐ (between 5 and 25 mm), and microplastics (MPs < 5 mm). Furthermore, there is a high possibility of further degradation and fragmentation of microplastics into nanoplastics (NPs), termed when the particle size ranges between 1 and 1000 nm (EFSA, 2016; Hartmann et al., 2019; Huang, Song, et al., 2021; Toussaint et al., 2019), due to environmental weathering and biodegradation. However, the lack of international consensus on these definitions causes ambiguous communication and non‐comparable data in scientific literature (Hartmann et al., 2019). Currently, there is still some discussion about overlapping size ranges between nano‐ and microplastics. Since there is little to no data on the interactions of NPs with the human body, we mainly refer to MPs for the purposes of this review. The combined term micro(nano)plastics (MNPs) will be used in general statements. Considering their origin, particles can be classified into primary MNPs, when intentionally manufactured (consumer products), or secondary MNPs, when released into the environment from slow fragmentation/degradation of larger plastics (Hartmann et al., 2019; SAPEA, 2019).

Most plastic particles are petroleum‐derived, such as polypropylene (PP), polyethylene (PE), polyvinyl chloride (PVC), polyethylene terephthalate (PET), and polystyrene (PS) (Geyer et al., 2017; Paul et al., 2020). Currently, the dominant polymer types are fossil fuel–based plastics and less than 1% are biodegradable; and of the almost 370 million tons of plastic produced annually, only a small fraction (≈1%) is bio‐based (European Bioplastics, 2021).

Because of their inherent characteristics, especially their high‐molecular‐weight and high crystallinity, plastics degrade and accumulate in numerous forms in the environment (Gewert et al., 2015), which can trigger serious global pollution problems, adversely affecting organisms, soil, and water. Furthermore, plastics/MNPs contain different additives and can adsorb metals and persistent organic pollutants (Campanale et al., 2020). Moreover, because of their hydrophobic character, hard properties, and strong floatability, MNPs are potential vectors for microorganisms and/or pathogens in oceans and natural microbial environments (Mammo et al., 2020). These contaminants can be transferred to organisms and biological tissues after plastic ingestion, a fact recently verified, especially for plastics in MP form (Elizalde‐Velázquez et al., 2020; Huang, Song, et al., 2021). Initially, the effects of MNPs on marine biota were believed to be a marine pollution issue. However, over the last decade, MNP research has progressed rapidly with discoveries of MNPs in freshwater, snow, ice, air, and even ocean spray, whereas soil and terrestrial biota, correspond with the more recent research focus (Allen et al., 2022). MPs have now been found in every environmental ecosystem investigated, and within a very broad spectrum of marine and terrestrial species, including humans.

The main concern related to MNPs in the environment includes their potential entry into the food chain and diet, both for food security and human health risk assessment. However, it remains poorly understood whether daily amounts of MNPs entering human organisms may have an important role in human health and future community health and whether the interaction between MNPs and associated chemical/biological contaminants can cause biomagnification effects. Increased evidence of the existence of a bioaccumulation of MNPs in the digestive tract of different organisms, with the presence of MNPs detected in the feces of aquatic and high‐trophic‐level organisms (Huang, Weng, et al., 2021; Lu et al., 2019) as well as in human feces (Schwabl et al., 2019; Yan, Liu, et al., 2022; Zhang, Wang, et al., 2021), suggest the connection between MPs and gut human microbiota and its consideration to health, which needs to be examined and understood.

Besides considering adverse biological effects, researchers are also focused on microbial communities and gut microbiomes as potential future bio‐tools for the remediation of plastic waste (Wang et al., 2022; Yang et al., 2015). Only 21% of plastics are estimated to be recycled or incinerated; the rest go to landfills or enter the natural environment (Lau et al., 2020). In addition, the diversity of polymer types, surface contamination, and low density of post‐consumer material (s) further limit their capacity for recycling. Thus, different biotechnological solutions for plastic biodegradation involving microorganisms and their polymer‐active enzymes, as well as gut microbial communities, are garnering increasing interest. At this point, it is critical to evaluate whether intestinal microbiota (i.e., human gut bacteria) influences the composition and structure of ingested MNPs, which may modify and determine the characteristics of the particles, changing their ultimate biological effects (Fournier et al., 2021; Wen, Zhao, Wang, et al., 2022).

Although there have been recent reviews on the interaction between plastics and environmental microorganisms and microbiomes (Amobonye et al., 2021; Lear et al., 2021; Santos et al., 2022), there are only a few reports from the viewpoint of the gut microbiome and the potential risk of these interactions for human physiology and health. Therefore, this review, after illustrating major advances and knowledge on dietary MNP‐exposure, aims to integrate current research on MNPs and their interaction with the gut microbiota at two levels: (1) considering the effect of food‐derived MNP particles on the gut microbiota within the gastrointestinal tract and on human health and (2) describing the possible effect of the gut microbiota on MNP biotransformation, highlighting the putative consequences for humans.

## FOCUS ON HUMAN HEALTH IMPLICATIONS: THE NEED TO INVESTIGATE MNP–HOST GUT MICROBIOTA INTERACTIONS

**FIGURE 1:**
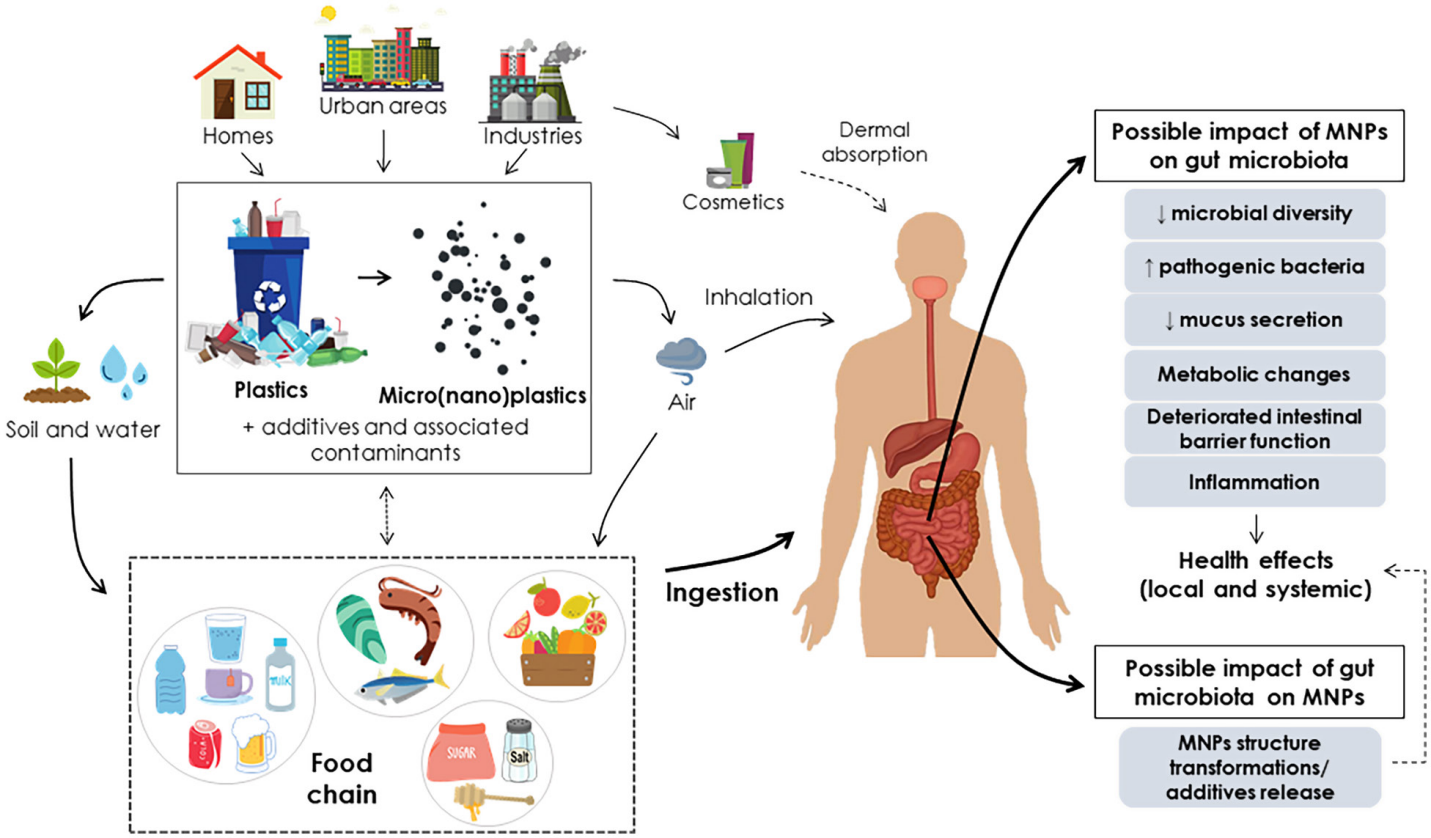
Routes of micro(nano)plastic exposure to humans and their impact on the gut microbiota. Designed using elements by ©Canva via Canva.com (access date: May 2022, version used Canva 2.0)

Although environmental toxicology research of MNPs has been ongoing for some time, human health toxicology studies have only recently been initiated. Three main routes of exposure to MNPs have been proposed: inhalation, ingestion, and dermal absorption, although the first two are the most notable routes of exposure (Figure 1). The latest evidence of animal and human exposure to MPs and NPs reinforces the current concern about these particles as food contaminants. The detection of MPs in animal and human feces has confirmed the oral route (Schwabl et al., 2019; Zhang, Li, et al., 2021), suggesting that plastic particles are ingested directly or with food and beverages and could be susceptible to changes during digestion (Xu et al., 2022). Therefore, there is growing interest in quantifying the real exposure to MNPs and their health effects, considering both comprehensive human consumption and internal exposure, including the interaction of the different MNPs with the gut microbiota.

### Human exposure to MNPs via the food chain and drinking water

Besides the first investigation that showed the presence of MPs in food for human consumption (Fendall & Sewell, 2009), numerous studies have revealed the presence of plastic particles throughout the food chain and drinking water, as well as supported human interaction with MNPs via ingestion. Although the use of plastics as food packaging materials has increased, all plastic materials that meet food must be sufficiently inert to avoid the transfer of molecules that could alter the composition and organoleptic characteristics of food (Fasano & Cirillo, 2018; Serrano et al., 2014). Therefore, plastic particles that contaminate the food chain can have other origins, where several ways of contamination have been postulated: (i) MNPs can be directly ingested by marine and terrestrial organisms and absorbed by plants because of their small size, thus entering the food chain, (ii) raw materials could be contaminated, such as water, (iii) because the presence of MNPs in the air has been demonstrated, part of these particles could be deposited on food during its processing, storage, transport, or packaging (Jin et al., 2021; Toussaint et al., 2019; Wieland et al., 2022). To date, and although it is an expanding field, few studies have tried to precisely quantify MPs and NPs in food and beverages (Danopoulos, Twiddy, et al., 2020; Pivokonský et al., 2020; Toussaint et al., 2019; Wang, Lin, & Chen, 2020), and the available information is mainly limited to a few polymers, sizes, shapes, and exposure concentrations (Table 1). Because the first investigations of contamination by MPs were conducted in the sea, most of the scientific evidence includes marine products, such as fish, mollusks, or crustaceans (Barboza et al., 2018; Kwon et al., 2020; Walkinshaw et al., 2020), followed by salt, bottled drinking water, and other beverages, sugar, honey, fruits, and vegetables (Table 1). In contrast, only a few studies have attempted to estimate the total amount of MPs ingested through the diet. For example, Cox et al. (2019) estimated the consumption of 39,000–52,000 particles/year based on age and sex, considering only 15% of the caloric intake of Americans. Furthermore, if water consumption was only from bottled water, the figure would increase by 90,000 particles/year, compared to 4000 particles/year if only tap water was consumed. More recently, Senathirajah et al. (2021) have estimated an average consumption between 11,484 and 193,200 particles/year per person, which is equal to 0.1 to 5 g of MPs per week. Furthermore, based on the levels of MPs published contained within drinking water, crustaceans and mollusks, fish, and salt, and using the mean European dietary consumption, Rubio‐Armendáriz and colleagues estimated the tentative MPs/day exposure assessment; the intake of 2 L/day of water, 70.7 g/day of crustaceans/mollusks, 70.7 g/day of fish, and 9.4 g/day of salt would generate a maximum exposure to 33,626, 212.0, 409.9, and 6.4 particles of MPs/day, respectively (Rubio‐Armendáriz et al., 2022). However, because numerous limitations hinder the comparison of results, both exposure values and the concentration of MPs in food and beverages should be considered with caution (Brachner et al., 2020; Rubio‐Armendáriz et al., 2022). Complicated sources of plastics lead to diverse forms of MNPs in polluted food matrix. So, technologies for precise quantitative characterisation are needed, especially more advanced ones for nano‐sized plastics. Methods of sample collection and analysis are rapidly evolving; however, a standardization of analytical methods and controls is required, including a consensus on the definition, description, and expression of the results. Furthermore, MNPs reaching the food chain can absorb biomolecules and other materials from their environment (natural matter, chemical contaminants, and pathogenic microorganisms) known to enhance cellular uptake (Ramsperger et al., 2020). Another issue is that dietary MNPs will be subjected to technological processing treatments during food production or cooking before ingested, which could alter the number and characteristics of plastic particles that come into contact with the human body (Wen, Zhao, Wang, et al., 2022). Finally, although concern over food contamination by MNPs is evident and government agencies have called for assessment of human health risks from exposure to plastics from ingestion, there are no food consumption regulations currently in place regarding exposure to plastics or any other regulations regarding plastic exposure.

**TABLE 1:** Concentration, size, and type of micro(nano)plastics detected in various food and beverages.

Type of food/beverage	MNPs concentration	MNPs size	MNPs type	Identification method	References
Beverages					
Water					
Tap	0–1247 MNPs/L	<1 μm	PET, PP	FTIR, Raman spectroscopy, Pyr‐GC–MS or SEM–EDX	Danopoulos, Twiddy et al. (2020)
Bottled	0–1.1 × 10^8^ MNPs/L	<1 μm	PET, PP		
Soft drinks	0–7 MPs/L	100–3000 μm	PA, PEA	Epifluorescence microscopy, SEM–EDX, μRaman spectroscopy	Shruti et al. (2020)
Energy drinks	0–6 MPs/L	100–3000 μm	PA, PEA		
Cold tea	1–6 MPs/L	100–3000 μm	PA, PEA		
Beer	0–28 MPs/L	100–3000 μm	PA, PEA, PET		
Milk	204–1004 MPs/100 ml	≥ 5 μm	PE, PES, PP, PTFE, PS	μRaman spectroscopy, SEM–EDX	da Costa Filho et al. (2021)
White wine	2563–5857 MPs/L	26 × 122 μm	PE	Optical microscopy, μRaman spectroscopy	Prata et al. (2020)
Food					
Seafood					
Molusks	0–10.5 MNPs/g	<5 μm	PE, PP, PET	FTIR, Raman spectroscopy, Pyr‐GC–MS or SEM–EDX	Danopoulos, Jenner et al. (2020)
Crustaceans	0.1–8.6 MNPs/g	Unknown	PE, PA, PP, PET		
Fish					
Fresh	0–2.9 MNPs/g	Unknown	PE, PP, PET		
Canned	0.05–0.22 MPs/g	10–1100 μm	PET, PS, PP	SEM–EDX, μRaman spectroscopy	Akhbarizadeh et al. (2020)
Meat					
Poultry	0–2.9 MPs/g	3–4000 μm	PS, PVC	ATR‐MIR	Huang et al. (2020)
Eggs	11.67 ± 3.98 MPs/egg	50–100 μm	PES, PP, PTFE, PS, PA, PU, PSU, PVA	Fluorescence microspy, ATR‐FTIR	Liu, Chen, et al. (2022)
Table salt	20–125 MPs/kg	20–3000 μm	PE, PP, PET, PS, PVC, PA, PMMA, PC	μ‐FTIR, Py‐GC/MS	Li, Wu, et al. (2022)
Honey	32–108 MPs/kg (fibres)	–	Celulosa, PET	Light microscopy, Raman spectroscopy, ATR‐FTIR	Mühlschlegel et al. (2017)
Fruits					
Apples	52,600–307,750 MPs/g	1.56–3.19 μm	Unknown	SEM–EDX	Oliveri Conti et al. (2020)
Pears	98,325–302,250 MPs/g	1.87–2.59 μm	Unknown		
Vegetables					
Broccoli	65,025–201,750 MPs/g	1.86–2.95 μm	Unknown		
Lettuce	26,375–75,425 MPs/g	2.18–2.78 μm	Unknown		
Carrots	72,175–130,500 MPs/g	1.36–2.00 μm	Unknown		
Sugar	32 ± 7 MPs/kg (fibres); 217 ± 123 MPs/kg (fragments)	Unknown	Unknown	Dissection microscope	Liebezeit and Liebezeit (2013)

Abbreviations: ATR‐FTIR, attenuated total reflection–Fourier transform infrared spectroscopy; ATR‐MIR, Attenuated total reflection mid‐infrared spectroscopy; FTIR, Fourier‐transform infrared spectroscopy; PA, polyamide; PC, polycarbonate; PE, polyethylene; PEA, poly(ester‐amide); PES, polyethersulfone; PET, polyethylene terephthalate; PMMA, poly(methyl methacrylate); PP, polypropylene; PS, polystyrene; PSU, polysulfone; PTFE, polytetrafluoroethylene; PU, polyurethane; PVA, poly(vinyl alcohol); PVC, polyvinyl chloride; Pyr‐GC–MS, pyrolysis gas chromatography–mass spectrometry; SEM–EDX, scanning electron microscopy and X‐ray microanalysis.

### The effects of MNPs on the gut microbiota seem multiple

Multicellular organisms have co‐evolved with complex communities of microorganisms (microbiota) and their genomes (metagenome), collectively referred to as microbiomes (Marchesi & Ravel, 2015). They develop symbiotic relationships that benefit both organisms. The human gut microbiome comprises representatives of bacteria—primarily species belonging to *Bacteroides* and *Firmicutes—*but also includes Archaea, lower and higher Eukarya, and viruses. Appreciation of the importance of gut microbiome–host interactions has grown over the past 20 years, with scientific findings supporting the key role of the gut microbiota in the appropriate host (both human or other animal) development with its implications in host physiology and health maintenance. Although the composition of an adult microbiota remains relatively stable, it is well known that microbial diversity is acquired very early in life within the first hours after birth and is shaped over time as the diet becomes more complex and the immune system matures (Cani, 2018). Therefore, the combination of multiple factors, including genotype, mode of delivery, early antibiotic therapy, diet composition, lifestyle, social interactions, and environmental exposure to various xenobiotics, shape the gut microbiota to make each individual microbially unique. In addition, the gut microbiota is considered an ‘organ’ with front‐line exposure to environmental changes and trauma. MNPs can enter the gut directly and accumulate in the intestine; thus, some animals consumed as whole organisms can represent an important vector for human consumption of MNPs. Moreover, the concomitant ingestion of MPs in the trophic chains associated with the diet can affect host intestinal microbial communities. The microbiota of a healthy individual is resilient, with the ability to quickly and completely return to baseline after a challenge, maintaining a dynamic equilibrium. However, when a disturbance or change becomes chronic, it can lead to a new altered stable equilibrium or a state of dysbiosis related to different diseases at the gastrointestinal and systemic levels (Lu et al., 2019; Thursby & Juge, 2017). To address this issue, the latest results of in vivo studies and in vitro gastrointestinal tract systems have been provided.

#### Changes in the microbiota due to MNPs in aquatic organisms and invertebrate models

Most studies analyzed that involved the effect of MNPs on intestinal microbiota were conducted in animal models, mainly zebrafish (*Danio rerio*) and mice. In adult and larval zebrafish, PS MP exposure was characterized by a change in the *Firmicutes/Bacteroidetes* ratio, whose increase has been associated with different diseases, such as obesity (Jin et al., 2018; Turnbaugh et al., 2006; Wan et al., 2019; Zhao et al., 2021). Furthermore, NPs have been reported to induce more severe dysbiosis and inflammation than MPs in zebrafish (Xie et al., 2021). In adult Medaka fish (*Oryzias melastigma*), Zhang, Wen, et al. (2021) detected an alteration of the gut microbial communities after exposure to PS MPs (2, 10 and 200 μm, spheres), depending on particle size. Moreover, the increase in proportions of *Verrucomicrobia* and *Firmicutes/Bacteroidetes* ratio and the decrease in *Fusobacteria* members were correlated with an increase in body weight. In common carp (*Cyprinus carpio* L.), PS MPs (32–40 μm) triggered changes in the abundance of pathogenic bacteria, such as *Shewanella*, *Plesiomonas*, and *Flavobacterium*, related to physical and chemical intestinal barrier dysfunction (Ouyang et al., 2021). Furthermore, the effects of exposure of untreated and seawater PS MPs on the gut microbiota of marine bivalve blue mussel (*Mytilus edulis*) have been reported at relatively realistic concentrations (0.2 mg/L, 32–40 μm, spherical); potential human pathogens had increased abundance induced by exposure to MPs for 6 weeks, and some retained higher abundance after 8 days depuration (Li et al., 2020). The effects on intestinal microbiota induced by MNPs may also be possibly because of the biofilm on their surfaces, which causes notable impacts on aquatic animals (Yan et al., 2021). Furthermore, a notably higher prevalence of antimicrobial‐resistant genes has been reported in microbiomes isolated from MPs than in seawater, highlighting the ability of MPs to act as habitats for increased gene exchange (Liu et al., 2021). Regarding metabolic activity, MPs alone or combined with other pollutants, such as glyphosate, triggered microbial metabolic changes in common carp, affecting differential metabolites related to amino acid and lipid metabolism (Chen, Rao, et al., 2022), which can be detrimental to essential functions and reproductive capacity for chronically exposed species (Fackelmann & Sommer, 2019; Galafassi et al., 2021). Because of a mutual link between the microbiota, the immune system, and the metabolome, MPs can directly or indirectly affect all three systems and therefore are challenging to decipher which system the MPs have the greatest impact on.

Regarding soil dwelling species, such as collembola (*Folsomia candida*), an increase in its gut microbiota alpha‐diversity and relative abundance of *Bacillaceae* was found after 56 days exposure to PVC particles (80–250 μm, round irregular shape) compared to non‐exposed counterparts. Exposure to MPs appears to impact collembolan feeding behavior through changes in their microbiota that lead to altered growth and reproduction (Zhu et al., 2018). On the other hand, PS MNPs (0.1, 10, and 100 μm, spheres) reduced the accumulation of metals (Ni and Pb) in earthworms (*Eisenia fetida*) associated with changes in bacterial community diversity by a size effect, especially featuring a higher relative abundance of *Proteobacteria* and *Bacteroidetes* than the control group (Yang et al., 2022). In insects such as bees, exposure to PS MPs (25 μm, spheres) decreased the alpha‐diversity within the gut microbiome and changed the structure of the core microbial population compared to control bees (Wang, Li, et al., 2021). Finally, the species *Caenorhabditis elegans* is the most studied nematode regarding the toxic effects of MPs. MP particles can interrupt the digestive tract of nematode species, leading to growth reductions, and these toxic effects appear to be exacerbated by chemical additives bound to plastic particles (Jewett et al., 2022). Interestingly, in *C. elegans*, fecal microbiota transplants attenuate small plastic–mediated toxicity (Chu et al., 2021), suggesting that some microbes play a protective role in our gut, which still needs to be verified at the mammalian level.

#### Microbiota changes due to MNPs in mice models

To date, no data have elucidated the impacts of MNPs on human gut microbiota in vivo; however, several studies already conducted in mammals revealed modifications of the microbial communities after short‐ and long‐term MNP ingestion, which can lead to changes in the balance of commensal populations, thus allowing the overgrowth of pathogens and pathobionts—commensal organisms can cause disease when specific genetic or environmental conditions are altered in the host. Our literature analysis revealed that PS spheres are the main MNPs studied, followed by PE. Furthermore, feces and cecum contents are the most analyzed gut microbiota samples. 16S rRNA gene‐based sequencing analysis of the cecal content of MNP‐treated mice demonstrated impaired α and β‐diversities; at the phylum level, it was characterized by changes in *Bacteroidetes*, *Firmicutes*, *Actinobacteria*, and *Proteobacteria*. At the genus level, changes in the abundance of *Staphylococcus*, *Clostridium*, and *Bacteroides* were detected compared to untreated animals. Moreover, up to 15 types of bacteria were affected after exposure to MPs, in particular, *Bifidobacterium*, *Prevotella*, *Veillonella*, *Actinobacteria*, and *Ruminococcus* (Jin et al., 2019; Lu et al., 2018). Regarding *Proteobacteria*, some discrepancies were shown, and depending on the study, a decrease or increase of its fecal relative abundance has been reported. Lu et al. (2018) and Jin et al. (2019) found a relative reduction in the abundance of *Proteobacteria* in ICR mice after treatment with 0.5, 5, and 50 μm PS MNPs at concentrations of 100 and 1000 μg/L. The same results were obtained from another mouse model (C57BL/6) and different dose of MPs (0.1 mg/day) by Chen, Zhu, et al. (2022). In contrast, Jiang, Yuan, et al. (2021) and Qiao et al. (2021) found an elevation in relative abundance of *Proteobacteria* in C57BL/6 mice after PS MP ingestion, which is consistent with the findings of Liu, Lv, et al. (2022). Gut microbiota is susceptible to physiological stress and live condition of the host, which may be the reason for the above discrepancy in gut microbial alterations detected in in vivo experiments with different mouse species, MNP properties, and exposure duration. The importance of particle size and charge in the toxicity induced by PS MNPs has recently been reported in mice. Oral exposition of three types of PS particles (PS, negatively charged carboxylated PS, and positively aminated PS spherical particles of two sizes [70 nm and 5 μm in diameter]) for 28 days caused gut tract injuries, leading to enriched opportunistic pathogen genera, accompanied by a deteriorated intestinal barrier function (Qiao et al., 2021). The micro‐sized PS particles exhibited a more notable impact on various gut genera than their nanosized counterparts. Some recent studies have further postulated that the uptake and effects of PS MPs and NPs in mice could depend on the exposure time, as shown with short‐ or medium‐term exposure (up to 42 days) (van Raamsdonk et al., 2020). Chronic exposure to PVC MPs (60 days, 2 μm, round irregular shape) and PS MPs (90 days, 5 μm, spheres) decreased the relative abundance of commensals (including *Muribaculaceae*, *Prevotellaceae*, and *Enterorhabdus*) and affected the abundance of conditionally pathogenic bacteria (*Blautia*, *Staphylococcus*, *Parasutterella*, and *Mucispirillum*) in adult mice (Chen, Zhuang, et al., 2022; Wen, Zhao, Liu, et al., 2022). Changes in commensal bacteria led to a decreased bile acid content, which is related to disordered lipid metabolism (Chen, Zhuang, et al., 2022). Wen, Zhao, Liu, et al. (2022) conducted a fecal microbiota transplantation trial to ulteriorly demonstrate the critical role of altered gut bacteria from MPs in liver susceptibility and hepatoxicity. Furthermore, several studies investigated the effects induced by MPs containing environmentally relevant additives. For instance, Deng et al. (2020) valuated the co‐exposure of PE MPs (45–53 μm, spheres) and di‐(2‐ethylhexyl) phthalate (DEHP) in CD‐1 mice during 30 days and found that gut microbiota disturbances were stronger for phthalate‐contaminated MPs than for pristine MPs. Besides changes in the bacterial community, MPs also influenced bacterial metabolism in in vivo rodent models. An increase in amino acid metabolism by the gut microbiota has been observed in mice exposed to PE MPs (1–10 μm, spheres) compared to non‐gavaged animals (Sun et al., 2021). Jin et al. (2019) and Qiao et al. (2019) also suggested the effects of PS MPs (5 μm, spheres) on the balance of fatty acids and glycolipid metabolism pathways, related to the survival and growth of bacteria.

As indicated above, current studies on mouse models still suffer from limitations; in particular, the administrated dosage of commercial MPs (converted to weight/kg/day), greatly varies in the revised studies, ranging from 15 μg/kg/day to 100 mg/kg/day. The exposure time in tests with microbiota also varies in the revised studies. It is foreseeable that realistic concentrations and longer exposure periods will be tested in future trials as they represent a more environmentally relevant approach. MNP realistic shape is also a key issue. However, despite these methodological gaps, early studies suggest MNP exposure to be detrimental for gut ecosystems and gut homeostasis in vivo. These data call for research on the effects of accumulated MPs on the human microbiota. Moreover, future research will need to focus on the potential effects of MNPs on the diverse consortium of bacteria, archaea, fungi, protozoa, and viruses, that inhabit the gut of all mammals, as well as the corresponding mechanisms regarding how the microbiome impacts MNP toxicity effects.

#### Physiologically relevant in vitro models of the human gut to understand the interaction of MNPs with intestinal contents

Although there is a lack of literature related to humans, the presence of MPs in infant and adult human feces provides evidence for its critical impact on the digestive tract (Schwabl et al., 2019; Yan, Liu, et al., 2022; Zhang, Li, et al., 2021; Zhang, Wang, et al., 2021). Animal models serve as a first approximation to study the gut ecosystem; however, because of their clear physiological differences from humans, the results are not entirely transferable; thus, in vitro gastrointestinal models are another strategy for obtaining consistent evidence. These tools mimic or simulate the physiological conditions that occur during the phases of human digestion, so they serve as a valuable alternative to animal models and human studies when they are not viable for ethical reasons (Fournier et al., 2021). To date, only three studies have conducted static digestions of MPs (Huang, Yin, et al., 2021; Stock et al., 2020; Tan et al., 2020), of which only Huang, Yin, et al. (2021) simulated microbial colonic fermentation. Their results showed that PE MPs (5, 10, 25 and 50 mg/L, 30–140 μm) increased the proportions of *Clostridium*, *Bacteroides*, and *Escherichia* in the gut microbiota. Furthermore, both PE and tetrabromobisphenol A, a plastic additive, transformed the composition of the microbiota and the metabolism pathways, altering gut homeostasis (Huang, Yin, et al., 2021). Regarding dynamic multi‐compartmental models, of the few studies available (Godoy et al., 2020; Tamargo et al., 2022; Yan, Zhang, et al., 2022), Tamargo et al. (2022) were among the first to evaluate the effects of MPs on human fecal bacteria. Simulating the entire gastrointestinal tract (stomach, small intestine, and three parts of the colon) by combining a harmonized static model and the dynamic gastrointestinal simgi® model, they showed that PET MPs (166 mg/intake, 160 ± 110 μm, irregular shape) reduced biodiversity and altered the composition of the colonic microbiota, possibly due to the attachment of some colonic microbiota to MP surfaces. Different types of bacteria were affected, in particular a decrease in beneficial bacteria as *Christensenellaceae* was observed, as well as an increase in the proportions of *Escherichia/Shigella* and *Bilophila*, often associated with a pro‐inflammatory effect in the gut (Tamargo et al., 2022). More important, the same authors described a bidirectional interaction between MPs and the microbiota for the first time. MPs tended to change intestinal microenvironments and affected bacterial growth and composition, whereas some members of this community could be related to PET MP biotransformations in the gut and thereby altering its bioavailability or toxicity (Tamargo et al., 2022). Furthermore, using a mucosal simulator of the human intestinal microbial ecosystem (M‐SHIME), Yan, Zhang, et al. (2022) investigated the differential effects of PET MPs (2 g/day, distribution of sizes with ≈80% between 100–300 μm, irregular shape) on the intestinal luminal microbiota and the mucosal microbiota. These two microbial niches harbor different microbial compositions and functions. Microbes significantly changed because of treatments, and MPs induced stronger effects on luminal microbiota than specifically mucosal microbiota (Yan, Zhang, et al., 2022). As the authors indicated, the possible reason is that mucus and mucin provide many attachment sites for intestinal microbes, promoting their colonization and stabilization. Furthermore, co‐exposure of MPs and phthalates caused aggravated effects on human fecal microbiota and altered its metabolic function. In particular, MP exposure reduced the production of short‐chain fatty acids (SCFAs)—the main metabolites produced by bacterial colonic fermentation, which have a key role in host metabolism and inflammation.

These emerging studies indicate that relevant gut systems that offer complexity similar to that in vivo, under standardized approaches, offer promising opportunities for investigations related to MPs in the complex gastrointestinal environment. Likewise, they can simulate long‐term/repeated exposure of MPs in interactions of the human intestinal microbiota due to the consumption of contaminated food and aid elucidating mechanisms and physiological behavior. Although studies in humans are a priority, the models being used are being validated with in vivo data; this bodes well for the future.

#### Known and potential factors and effects of MNPs related to gut microbiota disorders

MPs can remain in the colon, leading to long‐term and low‐level inflammation (Jin et al., 2018). Disruption of the gut microbiota will lead to increased gut barrier dysfunction and increased gut permeability and immunotoxicity, as reported in vertebrates and invertebrates (Hirt & Body‐Malapel, 2020) including in mammals (Djouina et al., 2022). Subsequently, gut bacteria and their products can enter the systemic circulation to cause damage to tissues and organs. Depletion of gut barrier integrity caused by opportunistic pathogens allows pro‐inflammatory metabolites, such as bacterial lipopolysaccharide (LPS), to pass through the gut, thus initiating proximal injury in other organs (Li, Lu, et al., 2022; Wen, Zhao, Liu, et al., 2022).

Mucus is the first layer in the gastrointestinal tract that foreign particles interact with. Among the factors that influence the mucus barrier, the microbiome plays an important role in driving mucus changes. Intestinal bacteria colonize the mucus layer using mucus‐derived nutrients and interact with the mucus layer (Paone & Cani, 2020). Intestinal dysbiosis can change the thickness of the mucus layer and could cause abnormal mucus invasion and epithelial adhesion of pathogens, or may even allow MNPs to interact directly with the epithelial layer and destroy the gut epithelium, changing the intestinal microenvironment (Huang, Weng, et al., 2021). Mucus‐associated bacterial biofilms could play a role in these disorders. According to limited studies in vivo, MPs remain attached to the intestinal mucus layer and come into direct contact with the apical part of intestinal epithelial cells, leading to local inflammation and toxicity to the intestinal barrier (Hirt & Body‐Malapel, 2020). Other studies have highlighted the possibility that MPs could act indirectly as carriers of potential biofilm‐associated opportunistic pathogens and antibiotic resistance genes in the human gut (Kirstein et al., 2016; Lu et al., 2019). Furthermore, the possibility of acting as a vector of fungi and viruses has been suggested (Vethaak & Legler, 2021). However, whether MPs and their adherent microorganisms compete for resources within the gut remains unexplored. In addition, the relationship between mucus structure changes and disease damage needs further study.

Regarding the translocation of MNPs from the gastrointestinal tract to the circulatory system and other body compartments, preliminary studies using human cells and rodents showed MNP systemic exposure by biodistribution and accumulation in different organs, including the liver, kidney, placenta, and brain (Grodzicki et al., 2021; Kwon et al., 2022; Mu et al., 2022; Prüst et al., 2020; Ragusa et al., 2021). Small plastic particles (<10 μm) may suffer gut epithelial absorption and systemic biodistribution to organs after exposure (Sun et al., 2022). Surface charges also play a predominant role in cell death induced by the interaction of MNPs with intestinal cells, suggesting that MNPs' different properties may undergo different internalized pathways that lead to diverse toxic effects (Banerjee & Shelver, 2021; Qiao et al., 2021). Internalization through M‐cells and paracellular persorption in the intestine are the most likely mechanisms underlying MNP uptake (Rubio et al., 2020). Such cells belong to mucosa‐associated lymphoid tissues and transport large structures (antigens, bacteria, and viruses) to the immune system. However, due to the complexity of the in vivo environment and limited analytical/methodological standardizations and lack of comparability between studies and treatment conditions (cell line or animal model used, experimental design, duration, doses, etc.), evidence on the efficacy of the intestinal barrier in translocation of different physicochemical attributes of particles and shapes is limited and controversial (DeLoid et al., 2021; Rodrigues et al., 2022; Sun et al., 2022; Visalli et al., 2021). Moreover, most studies reported so far focus on pristine particles, without considering the impact of digestive processes and/or the influence of chemical or microbial contaminants. The exact routes of MNPs' cellular intake, the tissue accumulation of MNPs, and the potential adverse effects after MNPs' long‐term exposure in humans are unknown. The fate and transport of MNPs upon entering an organism through absorption and excretion are also unclear. Plastic particles (≥700 nm) are bioavailable for uptake into the human bloodstream (Leslie et al., 2022). The urine excretion of MNPs seems slow, although NPs were excreted through urine in mice (Sun et al., 2022; Zhang, Wang, et al., 2021). A recent study to explore the pharmacokinetic profiles of acute and chronic exposure of PS MPs and NPs in mammals, revealed that most of the plastics (even the smallest particles, 20 nm) remain in the gastrointestinal tract and are eliminated through the feces by 48 h post‐ingestion, a result reinforced by acute biodistribution (Keinänen et al., 2021). Because feces are the main excretion pathway of MNPs of larger size, it is suggested that the intestine is not only a primary target organ but also may be an ultimate target organ; therefore, the effects on intestinal health need to be focused.

As the microbial genome confers metabolic capabilities exceeding those of the host organism alone, making the gut microbiome an active participant in host physiology, the potential biological/clinical consequences associated with MNP–microbiota interaction deserves much attention. Despite the scarcity of reports directly relevant to humans, evidence of a positive correlation between fecal MP concentration and the severity of intestinal inflammatory disease activity has recently been reported in a cohort of patients with inflammatory bowel disease (IBD) (Yan, Liu, et al., 2022). Higher amounts of different types of MPs were found in the feces of these patients, indicating that induced disturbances on critical intestinal functions by ingested MPs, such as microbiota alterations, could contribute in the long term to the onset of immune‐mediated inflammatory diseases in humans. Furthermore, changes in the microbial communities induced by exposure to MPs could affect physiological homeostasis, contributing to disease susceptibility in other organs, most likely cardiovascular and metabolic disorders, inflammation, and neurological diseases. For example, pre‐consumption of MPs predisposed chikungunya virus infection to a rise in fecal *Firmicutes/Bacteroidetes* ratio, resulting in prolonged viral arthritis in mice (Rawle et al., 2022). Susceptibility to obesity is critically linked to gut microbial imbalance, and among the mechanisms of the potential obesogenic action of MPs, induction of epigenetic changes in fat tissue and induction of gut microbiome dysbiosis have been reported (Kannan & Vimalkumar, 2021; López de Las Hazas et al., 2022). In a recent proof‐of‐concept study, MP contamination in liver samples from patients suffering cirrhosis was evaluated; an eight‐fold increase in plastic contamination in patients with liver disease compared to blank and liver samples from healthy individuals was found (Horvatits et al., 2022). The impact on the organs that first come into contact with ingested particles, the gut and liver, was further evidenced in mice, where liver malfunction was linked to gut microbiota (Wen, Zhao, Liu, et al., 2022). Likewise, MNPs are also likely to penetrate the blood–brain barrier, accumulate in the brain, and manifest neurotoxicity as recently evidenced by Zaheer and colleagues (Zaheer et al., 2022). As there is communication between the intestine and the brain, the authors reported a link between PE MP daily exposure during the prenatal and early postnatal periods and the development of autism spectrum disorder (Zaheer et al., 2022). Finally, as inhalation is another relevant source of MNP exposure, the combined study of MNP impacts on nasal and intestinal microbiota is another concern for human health, both in general public and high‐exposure population (Zhang et al., 2022).

The possibility that MNP‐induced dysbiosis leads to changes in metabolites and metabolism of the human microbiota, and its consideration as an indirect mechanism of MNP‐gut microbiota toxicity, is another important issue not previously considered. Thus, studies of gut microbiota–mediated modification of MNPs may give us new insights regarding the biological activity of MNPs when studying their adverse effects.

## LINKING THE GUT MICROBIOME AND MNPS' BIOTRANSFORMATION

MNPs have a high surface‐area‐to‐volume ratio that supports organic matter adsorption and represents a new habitat for diverse microbial assemblages, often referred to as the ‘Plastisphere’ in environmental sciences (Zettler et al., 2013). Although studies on MP colonization have focused mainly on the marine environment, where microorganisms can colonize the particles in minutes or hours, it has also been detected in terrestrial and atmospheric environments (Wang, Peng, et al., 2021). Furthermore, some studies have suggested that the colonization process follows a sequential taxonomic order: γ‐*Proteobacteria* members are predominant during the first stages, followed by α‐*Proteobacteria* (Wang, Peng, et al., 2021), generating a biofilm on the plastic surface over time, whose microbial communities appear significantly different from the surrounding environment (Zettler et al., 2013). Furthermore, there is an enrichment of plastic‐degrading bacteria species in these biofilms, including members of *Actinobacteria*, *Bacteroidetes*, and *Proteobacteria* (Zhang et al., 2019). Properties of plastic particles that make them inert for biodegradation include their hydrophobic nature, high‐molecular‐weight, and long polymer chain (Zhou et al., 2022); however, several studies have shown that some microorganisms ingest these polymers and convert them into environmentally friendly carbon compounds. Within this framework, microbiologists have tried to identify plastic‐active enzymes to implement them in industrial processes and in nature. These aspects have been comprehensively reviewed elsewhere (Chow et al., 2022; García‐Depraect et al., 2021). Today, the main challenge microbiologists are currently facing is finding polymer‐active enzymes targeting most fossil‐fuel–based plastics. In addition, identifying plastic‐active enzymes to implement them in biotechnological processes or understand their potential role in nature is an emerging research field (Chow et al., 2022). Here, we summarize the current knowledge on the microbial degradation of plastics by different microorganisms, with a special emphasis on gut microbiota, to address the question, if and to which extent intestinal microbes can alter/transform ingested MPs in the human gut.

### Environmental plastic‐degrading microorganisms

Polymer biodegradation is caused by microorganisms belonging to the three domains of life (Bacteria, Archaea, and Eukarya), but species from fungi and bacteria kingdoms are the most important players in the biodegradation process in natural environments. The type of plastic and the environmental conditions determine the most effective group of microorganisms, facilitating the degradation of the polymer (García‐Depraect et al., 2021; Maity et al., 2021). The bacteria most studied so far for their ability to degrade different plastics are members of the genera *Arthrobacter*, *Bacillus*, *Micrococcus*, *Pseudomonas*, *Corynebacterium*, *Streptomyces*, and *Nocardia* (Amobonye et al., 2021; Jacquin et al., 2019; Lear et al., 2021). *Ideonella sakaiensis*, a Gram‐negative rod‐shaped bacterium able to not only break down PET but also use PET as the sole carbon and energy source, has been a subject of numerous studies (Amobonye et al., 2021; Danso et al., 2019). Some fungi such as *Fusarium* spp., *Aspergillus* spp., and *Penicillium* spp. have also been described (Amobonye et al., 2021; Lear et al., 2021; Priya et al., 2021). Biodegradation pathways may depend on the microorganisms involved. For example, some of the most persistent types of MNPs in food and beverages such as PET and PP can be biodeteriorated and fragmented by certain species belonging to *Acinetobacter*, *Nocardia*, *Thermobifida*, *Pseudomonas*, or *Brevibacillus*. Biodegradation environments considered in most studies include composting facilities, marine environments (including the seawater–sediment interface), anerobic digestion facilities, aerobic freshwater environments, soil, and landfills (García‐Depraect et al., 2021). The basic steps of microbial degradation of both traditional and biodegradable polymers include biodeterioration, biofragmentation, and microbial assimilation and mineralization by aerobic or anerobic microbial species (García‐Depraect et al., 2021; Maity et al., 2021). A wide and suitable repertoire of extracellular genes, proteins, enzymes, and microbial metabolic pathways can alter plastic polymers and allow the depolymerization process; oxidases, amidases, laccases, hydrolases, and peroxidases are the main groups of microbial enzymes responsible for the degradation of polymers to monomers (Danso et al., 2019; Othman et al., 2021; Zhou et al., 2022). Each enzyme has a unique interaction mechanism, divided into two groups: enzymes that modify the surface of MPs by increasing their hydrophilicity (mainly hydrolases, lipases, carboxylesterases, cutinases, and proteases) and enzymes capable of degrading the internal areas of MPs, as with some cutinases (Othman et al., 2021). Although many reports have been published describing microbial communities that metabolize xenobiotics, highly active enzymes for most plastics, especially acting on high‐molecular‐weight human‐made polymers (which represent over 80% of annual plastic production) remain poorly identified (Carr et al., 2020; Danso et al., 2018). Until now, this research has largely failed to deliver functional biocatalysts acting on the commodity polymers such as PE, PP, PVC, and PS. Moreover, few enzymes are known to act on low‐density and low‐crystalline (amorphous) PET and and ester‐based PUR (Chow et al., 2022). Furthermore, the biochemical and structural properties of most of these enzymes and the different factors that affect the biodegradation of plastic are poorly understood. Although most studies highlighted the biodegradability of pure bacterial strains, in nature, bacteria often act synergistically in consortia. In addition, microbial species abundance and species diversity could affect the rate of biodegradation.

### Gut microbiota in the biodegradation of MNPs. Potential health impacts of plastics biotransformation by the gut microbiome

Besides free‐living microorganisms in the environment, gut microbiota is an important driver of MNP/plastic degradation, with most of the attention focused on insects and their larvae (Zhang et al., 2020). Some of the gut microbes that work in association with insects to degrade the most widely used plastics are highlighted in Table 2. Mealworm larvae (*Tenebrio molitor*) can degrade petroleum‐derived plastics such as PS, PP, PE, low‐density PE (LDPE), and PVC (Brandon et al., 2018; Peng et al., 2020; Yang et al., 2021), as well as bioplastic PLA (Peng et al., 2021). In fact, several studies have indicated this biodegradation does not occur after antibiotic treatment with mealworms (Yang et al., 2015, 2018) and that their gut microbiota changes after exposure to MPs, suggesting that their gut microbiome allows the degradation of different MPs (Bae et al., 2021; Brandon et al., 2018; Yang et al., 2021). Furthermore, wax moth larvae (*Galleria mellonella*) have also been studied, as they can degrade PS and PE, although it is not clear whether this ability depends only on the gut microbiota (Bombelli et al., 2017; Lou et al., 2020; Wang et al., 2022). Other species of larvae whose degradation process appears to be related to the gut microbiota are coleopterans, such as *Tenebrio obscurus* (Peng et al., 2019), *Zophobas atratu*s (Luo et al., 2021; Yang et al., 2021), *Tribolium castaneum* (Wang, Xin, et al., 2020), and *Plesiophthalmus davidi*s (Woo et al., 2020), as well as lepidopterans such as *Plodia interpunctella* (Yang et al., 2014) and *Achroia grisella* (Kundungal et al., 2019). Metagenomic analyses have revealed that *Proteobacteria*, as well as some *Firmicutes*, *Actinobacteria*, and *Bacteroidetes*, are predominant phyla present in the gut of diverse insect orders (Gambarini et al., 2021). Bacterial species that efficiently degrade plastics in laboratory studies belong mainly to *Pseudomonas* sp., *Bacillus* sp., and *Klebsiella* sp. (Jang & Kikuchi, 2020). These gut species mostly degrade plastic polymers by forming carbonyl groups via oxidation pathways, changing the chemical properties of plastic from hydrophobic to hydrophilic and depolymerizing the plastic. Inside the gut, MNPs are exposed to different enzymes that can also facilitate plastic degradation. However, unlike studies in the context of insects' gut bacteria and plastic bioremediation, little is known about the microbial degradation capacity of MNPs in mammals. This is probably because of the lack of appropriate high‐resolution analytical methods to detect and quantify small MPs and NPs and chemical intermediates in animal and human stools. The current references in laboratory‐based feeding studies in crustaceans, such as Antarctic krill (*Euphausia superba*) (Dawson et al., 2018), earthworms (*Lumbricus terrestris*) (Lwanga et al., 2016) and *Achantina fulica* snails (Song et al., 2020), revealed that these different organisms eventually disintegrate/fragmentate pristine PS, PE, and PET MPs into smaller pieces in their gut in natural environments. Biofilm formation has been shown to play a significant role in plastic bacterial decomposition because it promotes the adhesion of bacteria to the polymeric surface and their persistence (Puglisi et al., 2019; Santos et al., 2022; Wright et al., 2021). Regarding the human gut, feeding with PET MPs under simulated digestive conditions promoted the formation of biofilms that could favor the biotransformation of MPs through human fecal potentially degrading microbiota, suggesting that the human intestinal microbiota could harbor this degradation ability (Tamargo et al., 2022). Therefore, these studies suggest that common dietary‐MPs may be broken down in the gut into altered or even smaller nanometer‐sized particles that can be retained for some time in the digestive system. It is likely that bacteria use the plastic particle as a surface and ecological niche and degrade chemical additives if they become available (Wright et al., 2020). Regarding multispecies marine plastisphere, the combined action of some bacteria metabolites, such as organic acids, and different oxygen radicals (i.e., H_2_O_2_) within the plastic‐attached biofilms has been proposed as a starting point for the reactions involved in polymer breakdown (Chow et al., 2022). Thus, the toxicity of MPs for biofilms with the ability to favor disintegration of MPs in the gut requires more attention. Moreover, interactions between MPs and fecal/gut microorganisms over time in different environmental settings can be expected to define the microbial population and other contaminants on the surface of MPs. Thus, the importance of interactions between MPs and microbiomes, such as fecal/gut microbiome, deserves in‐depth mechanism studies from a health environmental perspective.

**TABLE 2:** Putative gut microorganisms responsible for the degradation activity of plastic particles observed in insects/larvae.

Organism	Plastic type	Gut microorganisms (family and genus/species)	Observations	References
*Tenebrio molitor*	PS	*Enterobacteriaceae* (*Citrobacter freundii*), *Spiroplasmataceae*, *Enterococcaceae*, *Rhodanobacteraceae* (*Dyella* sp.), *Xanthomonadaceae* (Lysobacter sp.), *Comamonadaceae* (*Leptothrix* sp.), *Rhizobiaceae* (*Agrobacterium* sp.), *Nitrosomonadaceae* (*Nitrosomonas* sp.), *Nitrospiriaceae* (*Nitrospira* sp.), *Streptococcaceae* (*Lactococcus* sp.), *Weeksellaceae* (*Elizabethkingia* sp.), *Listeriaceae* (*Listeria* sp.), *Hyphomicrobiaceae* (*Pedomicrobium* sp.), *Lamiaceae* (*Aquihabitans* sp.), *Yersiniaceae* (*Serratia marcescens*) and Proteobacteria (*Klebsiella* sp.)	Synergistic effect of the activities of larvae and their gut microbes. Mealworm may secrete emulsifying agents, increasing the bioavailability of PS and enabling more rapid microbial attack. Possible degradation by anerobic processes and diazotrophs	Peng et al. (2019), Przemieniecki et al. (2020), Brandon et al. (2021), Yang et al. (2021), Machona et al. (2022)
	PE	*Leptotrichiaceae* (*Sebaldella termitidis*), *Brevibacteriaceae* (*Brevibacterium* sp.), *Erwiniaceae* (*Pantoea* sp.), *Streptococcaceae* (*Lactococcus* sp.), *Weeksellaceae* (*Elizabethkingia* sp.), *Enterobacteriaceae* (*Citrobacter* sp. and *Kosakonia* sp.)	Possible degradation by larval esterases	Brandon et al. (2018), Przemieniecki et al. (2020)
	LDPE	Unknown	–	Wu et al. (2019)
	PP	*Enterobacteriaceae* (*Kluyvera* sp., *Citrobacter* sp., and *Enterobacter* sp.), *Streptococcaceae* (*Lactococcus* sp.), and *Spiroplasmataceae* (*Spiroplasma* sp.)	Depolymerization/biodegradation is gut microbe–dependent	Yang et al. (2021)
	PVC	*Streptococcaceae* (*Lactococcus* sp.), *Spiroplasmataceae* (*Spiroplasma* sp.), *Enterobacteriaceae*, and *Clostridiaceae*	Depolymerization/biodegradation was gut microbe–dependent	Peng et al. (2020)
	PLA	*Streptococcaceae* (*Lactococcus* sp.), *Spiroplasmataceae* (*Spiroplasma* sp.)	–	Peng et al. (2021)
*Tenebrio obscurus*	PS	*Spiroplasmataceae*, *Enterococcaceae*, and *Enterobacteriaceae*	Synergistic effect of the activities of larvae and their gut microbes	Peng et al. (2019)
*Galleria mellonella*	PS	*Bacillaceae* (*Bacillus* sp.), *Yersiniaceae* (*Serratia* sp.), *Enterococcus*, *Enterobacteriaceae*, and *Oxalobacteraceae* (*Massilia* sp. FS1903)	Collaboration between larvae and its gut microbiome in PS degradation	Lou et al. (2020), Jiang, Su, et al. (2021a, 2021b)
	PE	*Bacillaceae* (*Bacillus* sp.), *Yersiniaceae* (*Serratia* sp.), *Trichocomaceae* (*Aspergillus flavus* PEDX3) and *Enterobacteriaceae* (*Enterobacter* sp. D1)	–	Ren et al. (2019), Lou et al. (2020), Zhang et al. (2020)
	LDPE	*Moraxellaceae* (*Acinetobacter sp*.), *Bacillaceae* (*Lysinibacillus fusiformis*, *Bacillus aryabhattai*), *Microbacteriaceae* (*Microbacterium oxydans*), *Burkholderiaceae* (*Cupriavidus necator* H16) and *Pseudomonadaceae* (*Pseudomonas putida* LS46, *Pseudomonas putida* IRN22)	–	Bombelli et al. (2017), Cassone et al. (2020), Montazer et al. (2021)
*Zophobas atratus*	PP	*Enterobacteriaceae* (*Citrobacter* sp. and *Enterobacter* sp.) and *Enterococcaceae* (*Enterococcus* sp.)	Depolymerization/biodegradation is gut microbe–dependent	Luo et al. (2021), Yang et al. (2021)
	PS	*Enterococcaceae* (*Enterococcus* sp.), *Dysgonomonas*, and *Sphingobacterium*	Plastic degradation is associated with changes of gut microbial communities and digestive enzyme activities	Luo et al. (2021)
	PU	*Enterococcaceae* (*Enterococcus* sp.) and *Mangrovibacter*	Plastic degradation is associated with changes of gut microbial communities and digestive enzyme activities	Luo et al. (2021)
*Tribolium castaneum*	PS	*Moraxellaceae* (*Acinetobacter* sp.)	Plastic degradation is associated with changes of gut microbial communities and digestive enzyme activities	Wang, Xin, et al. (2020)
*Plesiophtalmus davidis*	PS	*Yersiniaceae* (*Serratia* sp.) and *Streptococcaceae* (*Lactococcus* sp.)	–	Woo et al. (2020)
*Plodia interpunctella*	PE	*Enterobacteriaceae* (*Enterobacter asburiae* YT1) and *Bacillaceae* (*Bacillus* sp.YP1)	–	Yang et al. (2014)
*Achroia grisella*	PE	Unknown	–	Kundungal et al. (2019)

Research connecting microbial degradation of MPs with microbiota in the human gut is still scarce, but many of the plastic‐degrading bacteria described in insects or larvae are part of the core human gut microbiota; notably, different potential pathogenic *Proteobacteria* such as species of the families *Enterobacteriaceae*, *Enterococcaceae*, *Listeria*, *Pseudomonas*, and *Klebsiella*, as well as other commensals such as *Lactococcus* (Oñate et al., 2019; Ruan et al., 2020). Members of the gut microbiome, especially those with potential pathogenic ability, such as *Proteobacteria* and other pathobionts, can adapt to hostile changes in environmental conditions and gain an advantage that competes with other members of the community. In this case, some members of the gut microbiome may have adapted to the accumulation of MPs in the gut and developed metabolic mechanisms and pathways to use these particles as a new carbon source to gain advantage over other microbial populations. Furthermore, such metabolic functions capable of degrading MPs into their monomers could facilitate their subsequent assimilation and degradation by other microbial groups. To date, there is no robust evidence to make accurate statements about this possibility nor about what kinds of enzymatic activities or microbial functions and plastic metabolites might be involved in the human gut. However, a recent study detected terephthalic acid (TPA, a PET monomer; 390–1600 ng/g) and bisphenol A (BPA, a PC monomer; 16–136 ng/g) in the feces of adults and infants, suggesting the connection between these plastic‐derived metabolites with biodegradation of PET and PC by the gut microbiota, respectively (Zhang, Wang, et al., 2021). Therefore, the combination of appropriate methods to assess changes in the structure of polymers and to identify potent microbial species (or consortia) and their fragmentation intermediates is crucial to understand the processes that lead to MPs being ingested.

Besides synthetic polymers, a variable percentage of the total weight of plastics is formed by a long list of additives that produce specific physicochemical properties for the desired final product. Consequently, considering that plastic degradation could also release different products depending on the type of polymer/additives and the conditions, some derived relevant additives and degradation products might negatively affect human health ‐formaldehyde, benzene, and furan, well‐known carcinogenic and mutagenic substances being the most dangerous (Amobonye et al., 2021; European Parliament and Council, 2008; Rodrigues et al., 2019). Additives that improve plastic characteristics could also harm human well‐being if released at the gut level. For example, plasticizers, like phthalates and bisphenol A, are known endocrine disrupting chemicals that can have hormonal activity that alters the homeostasis of the endocrine system (Campanale et al., 2020; Mathieu‐Denoncourt et al., 2015). Heavy metals, other plastic additives, and pollutants, plausibly susceptible to gut accumulation, are classified as probable human carcinogens based on evidence from epidemiological and experimental studies that have shown a correlation between exposure and cancer incidence in humans and animals (Tchounwou et al., 2012). The highest gastric/gastrointestinal bioaccessibility of MNP degradation products generated in the gut may pose severe risks to animals and humans. In particular, one of the most investigated plastic degradation products is para‐nonylphenol, whose suppressive effects on cell growth and physiological functions of several organisms have been linked to several diseases (Okai et al., 2022). Therefore, when addressing the possible effects that MNPs could have on our health, special attention should be paid to microorganisms that potentially release additives from MNPs because the adverse effects of these particles could be amplified. The human gut microbiota can extensively metabolize environmental chemicals (Claus et al., 2016; Koppel et al., 2017), which can also promote the release of additives. However, to the best of our knowledge, only the study conducted by Yan, Zhang, et al. (2022) has evaluated the release of additives (phthalates) in MPs by the gut microbiota. Using PET‐based single‐use beverage bottles as raw plastic, they found MPs can release different phthalates, specifically di‐(2‐ethylhexyl) phthalate (DEHP), di‐*n*‐butyl phthalate (DBP) and dimethyl phthalate (DMP), in a simulated gut environment, and the gut microbiota can accelerate such release; *Acidaminococcus* and *Morganella* were suggested as key colonic microorganisms correlated with the release of MP additives.

These preliminary evidences emphasize the importance of considering the human microbiota–MNP interactions that occur within our gastrointestinal tract regarding plastic structural changes and toxicity mechanisms. The products of gut microbial transformations can be absorbed by the host and circulated systemically or interact locally with the epithelial cells lining the gastrointestinal tract, most likely affecting both the host and the members of the microbiota. Given that the interplay between the gut microbiota and host cells is likely subject to high interindividual variability, these effects may have relevant implications for our ability to accurately predict a particular MP uptake and biodistribution in the body and a given population's response to MNPs.

## CONCLUSIONS AND PERSPECTIVES

Synthetic plastics are at the top of the list of ever‐accumulating pollutants, negatively affecting life on the planet. An annual production rate of 1100 tons of pristine plastic is expected by 2050; therefore, multidisciplinary research initiatives are urgently needed to support health policy decision‐making and mitigation strategies. Humans are exposed to MNP particles every day, and their intake through the food chain and drinking water represents a substantial source of exposure. The prevailing scientific data have shown that after oral exposure, MNPs have a negative impact on gut microbiota in a wide range of aquatic and terrestrial animal and mouse models, promoting intestinal dysbiosis, metabolic perturbed functions, and an inflammatory gut environment, as well as systemic effects in the host, of which the long‐term consequences are still unclear. The gut microbiota disruption can thus be an important biomarker for MNP toxicological assessment. In recent years, different examples of the impact of plastic degradation metabolism through bacteria and the gut microbiota of insects are gaining attention in the context of bioremediation. So far, there is no clear link between MNPs' microbial degradation and human gut microbiota, regarding specific ecological advantage and biological significance; however, additional studies in physiologically relevant advanced in vitro models suggest MPs suffer fragmentation and biotransformation during the digestive transit, which implies members of the human intestinal microbiota, and whose derived small particulate forms and released additives synergistically, might enhance MNPs adverse physiological effects. Therefore, deciphering the extent to which gut microorganisms diversify, MNP‐keystone species, specific mechanisms, and biological consequences will become important to understand our response to exposure of MNPs through diet. Although much more research is needed on human‐like conditions, increased in vitro and in vivo evidence implicates the gut microbiome as a key challenge for the connections between MNPs and human health, which also implies environmental health and its relationship to human habits. The gut microbiome affects the host and could also affect the bioaccumulation of MNPs in the human body; therefore, the extent of MNP ingestion and their metabolic fate must be evaluated, which requires detailed knowledge of the numerous kinds of plastics under realistic human life conditions, i.e., material composition (constituting polymers, additive cocktail, microbial pathogens, and toxins), size, shape, surface properties, exposure levels, and quantities, and finally, their ability to be absorbed in the gut and to cause systemic toxicity in the human body. It is expected that our understanding of the complex interconnectedness between MNPs, microbiome, and host will advance with new modeling systems, technology development, and refinement, and mechanistic studies focused on the contribution of human health and microbial metabolism and ultimately linked to sustainable food systems and planetary health.

## AUTHOR CONTRIBUTIONS

**Cristina Jimenez‐Arroyo:** Writing – original draft (equal); writing – review and editing (equal). **Alba Tamargo:** Writing – original draft (equal); writing – review and editing (equal). **Natalia Molinero:** Writing – original draft (equal); writing – review and editing (equal). **M. Victoria Moreno‐Arribas:** Conceptualization (lead); funding acquisition (supporting); supervision (lead); writing – original draft (lead); writing – review and editing (lead).

## FUNDING INFORMATION

The work in progress in our laboratory is partially supported by the Spanish Ministry of Science and Innovation (Spain), grant number PID2019‐108851RB‐C21, ALIBIRD‐CM 2020 P2018/BAA‐4343 (Community of Madrid) and by the European Union's Horizon 2020 Research and Innovation 772 program, under the Grant Agreement number 965367 (PlasticsFatE).

## CONFLICT OF INTEREST

We declare that we have no conflict of interest.
